# Clinical care given to victims of sexual assault at Kadoma General Hospital, Zimbabwe: a secondary data analysis, 2016

**DOI:** 10.1186/s12879-017-2702-4

**Published:** 2017-08-31

**Authors:** Stanley Tapesana, Daniel Chirundu, Gerald Shambira, Notion Tafara Gombe, Tsitsi Patience Juru, Tshimanga Mufuta

**Affiliations:** 1 Department of Community Medicine, University of Zimbabwe, Harare, Zimbabwe; 2 Kadoma City Health Department, Kadoma City, Zimbabwe

**Keywords:** Sexual Assault, Secondary Analysis, Clinical Care, Kadoma

## Abstract

**Background:**

Despite the guidelines for managing sexual assault being in place, victims of sexual assault attended to at Kadoma General Hospital consistently raised complaints related to the quality of care offered. Medicolegal data for sexual assault has been collected at the hospital since 2012. However, no analysis had been done regardless of complaints having been raised. We analysed the dataset to determine the quality of clinical care offered to sexual assault victims.

**Methods:**

A retrospective cross-sectional study based on secondary data was conducted. Epi. Info 7 software was used to analyse data and generate frequencies, measures of central tendency and proportions.

**Results:**

We analysed 474 medical affidavits completed between January 2014 and July 2016. Thirty percent of the victims sought care within 72 h of the sexual assault. Baseline HIV testing was done in 23 (22%) and follow-up HIV test done in 2 (2%) of the victims. Post Exposure Prophylaxis for HIV was administered to 18 (51%), emergency contraception 9 (69%) and forensic evidence gathered in six (5%) of victims presenting within the prescribed 72 h of the sexual assault. Prophylactic antibiotics were given to 156 (33%). There were no documented counselling sessions for all victims whilst follow up care was given to 47 (10%) victims.

**Conclusion:**

Suboptimal clinical care was given to victims of sexual assault during the period 2014-2016. These findings suggest possible delayed presentation by victims of sexual assault as well as suboptimal administration of prophylaxis by health care workers. We recommend adherence to guidelines in managing sexual assault. Further research to determine factors for delayed presentation among sexual assault victims and quality of care provided to them is recommended.

## Background

Gender-based violence (GBV) is a growing global public health concern of the twenty-first century. GBV cuts across all classes, races, ages, religions and national boundaries and contributes to social, legal and health challenges mainly affecting women [1–3]. Sexual assault is recorded as one of the worst forms of gender-based violence with long-term implications. The United Nations statistical report indicates that; globally more than 250,000 cases of sexual assault recorded annually. The prevalence of sexual assault ranges from 16.3% in East Asia to 65.64% in Central sub-Saharan Africa [4]. The estimated lifetime prevalence of sexual assault among primary students between 13 and 15 years in Sub-Saharan Africa ranges from 9% to 33%.

A variety of consequences of sexual assault on a survivor’s physical and mental health have been documented. These include physical injury, sexually transmitted infections (STIs) including HIV, unwanted pregnancy, unsafe abortion, anxiety, shame, posttraumatic stress, and depression [5]. In this regard, timely provision of health care to victims of sexual assault is crucial as several important aspects of the care are time dependent. The World Health Organisation guidelines for management of sexual assault covers the important aspects of management that include, provision of emergency contraception, post-exposure prophylaxis for HIV and/or collection of forensic evidence [6].

In Zimbabwe, management of sexual assault is guided by the Southern African Development Community Protocol on Gender and Development as well as the National Guidelines on Management of Sexual Assault [7, 8]. According to Zimbabwe Demographic and Health Survey (ZDHS 2015), 37% of those who experienced physical or sexual violence sought help [9].

Despite several complaints pertaining to the quality of care offered to victims having been raised, the medical affidavit dataset had never been analysed to determine the quality of clinical care offered to victims of sexual assault. We analysed the dataset in order to determine the adequacy of clinical care offered to sexual assault victims in line with the guidelines.

## Methods

### Study design and description of the study site

We conducted a retrospective cross-sectional study based on the secondary dataset at Kadoma General Hospital. Kadoma General Hospital is the only referral hospital therefore, all the sexual assault cases in Sanyati district are referred to this hospital.

### Study subjects and sample size

The study population was made up of all the medical affidavits completed at Kadoma General Hospital from January 2014 to July 2016. The study unit was a victim’s medical affidavit completed and kept at Kadoma General Hospital. Although the sample size was 357 medical affidavits we included all the 474 medical affidavits for analysis.

The variables that we investigated in our study included:

- **Socio-demographics-** age, sex, marital status**,** relationship to perpetrator, previous sexual experience and secondary sexual development characteristics
- **Time to presentation-** categorised as 0-24 h, 1-3 days, 4-7 days, 8-30 days, 1-3 months, > 3 months and unknown duration
- **Clinical evidence of sexual penetration-** categorised as definite, very likely, probable, inconclusive but possible and no visible evidence
- **Associated signs and symptoms-** behaviour change, genital discharge, genital pain, blood loss, dysuria, lower abdominal pain, difficulty in walking, genital sores, pain on defaecation, genital warts and other
- **Clinical management-** proportion of victims who received HIV Post-exposure prophylaxis, emergency contraception, prophylactic antibiotics, forensic tests, counselling and follow-up care
- **Laboratory tests done for victims-** baseline HIV test, follow up HIV test, vaginal swab microscopy, pregnancy test and syphilis test

### Data collection and analysis

Epi Info Companion (CDC, 2012) was used to capture data. The analysis was done using the same software. The software was used to generate frequencies, measures of central tendency and proportions. A sub analysis was done to assess the clinical care that was offered to the victims of sexual assault depending on their stage of development, prior history of sexual penetration, clinical presentation and clinical evidence of sexual assault.

### Ethical considerations

The names of health care workers, victims of sexual assault and police officers were kept confidential. This was achieved by assigning an anonymous identification number to each medical affidavit of sexual assault during data capture, and analysis. We obtained permission to proceed from the Provincial Medical Director Mashonaland West Province, Kadoma District Medical Officer and the Health Studies Office.

## Results

A total of 474 medical affidavits were analysed. The overall completeness of the dataset was 73%.

### Demographic characteristics

**Fig. 1:**
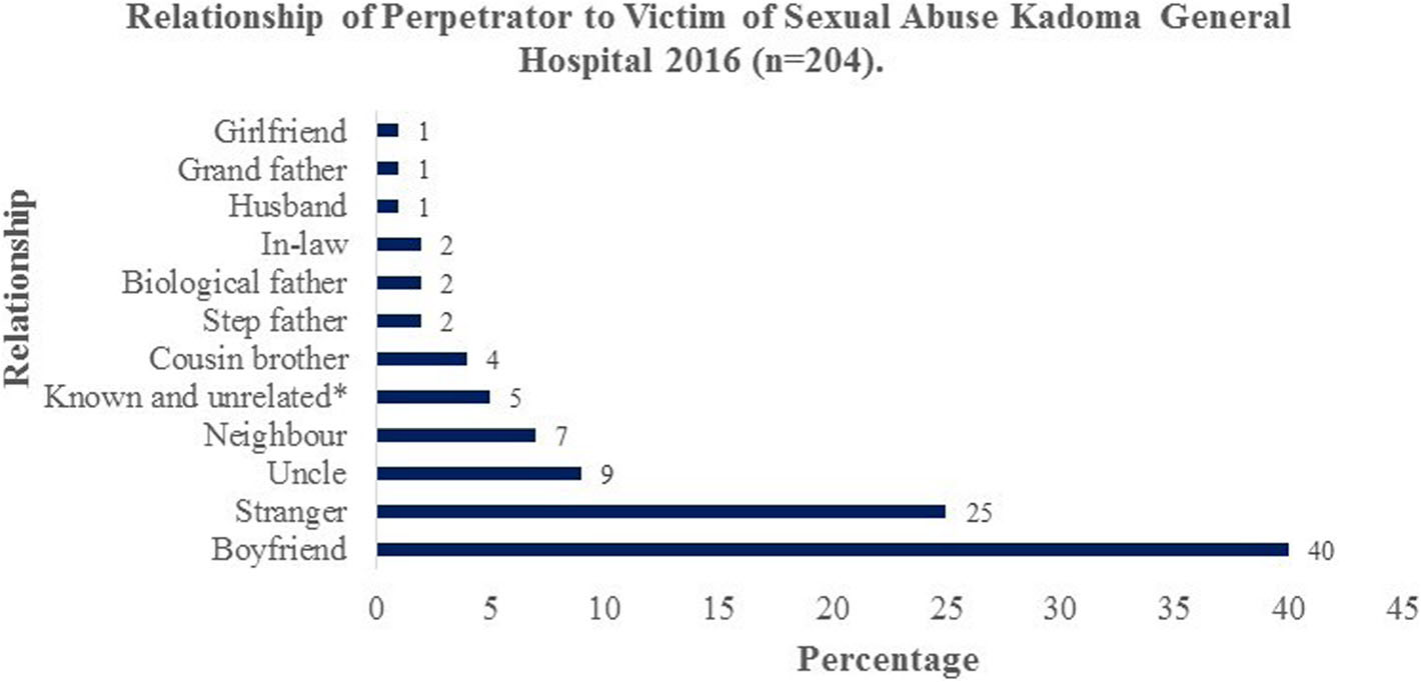
Relationship of the perpetrator to a victim of sexual assault Kadoma General Hospital 2016

The median age of the victims was 14 years (Q_1_ = 11; Q_3_ = 16), whilst that of perpetrators was 21 years (Q_1_ = 18; Q_3_ = 27). Four hundred and seventy-three (98.8%) of the victims were female. Seventy-four percent (204) victims of sexual assault demonstrated their relationship with perpetrators. Three hundred and thirty-one (72.6%) of the victims were younger than 16 years while 88(19.3%) were single women and 21(4.6%) married women. Male perpetrators were 204 (99.8%) as illustrated in Table 1. The relationship of perpetrator to the victim was that of a boyfriend in 81 (40%), stranger 51 (25%) and uncles in 18 (5%) as shown in Fig. 1.

**Table 1:** Demographic Characteristics of Sexual Assault Victims Kadoma General Hospital 2016 (*n* = 474)

Variable		n (%)
Sex of Victim	Male	1(0.2)
	Female	473(99.8%)
Relationship of victim to perpetrator	Related	150(74%)
	Not related	54(26%)
Marital status	Child under 16 years	331(72.59%)
	Single	88(19.3%)
	Married	21(4.61%)
	Divorced	13(2.85%)
	Other	2(0.44%)
	Widowed	1(0.22%)
Secondary sexual development	Pubertal	229(51.81%)
	Pre-pubertal	125(28.28%)
	Mature	86(19.46%)
	Post-menopausal	2(0.45%)

### Time to presentation and clinical evidence of penetration

**Fig. 2:**
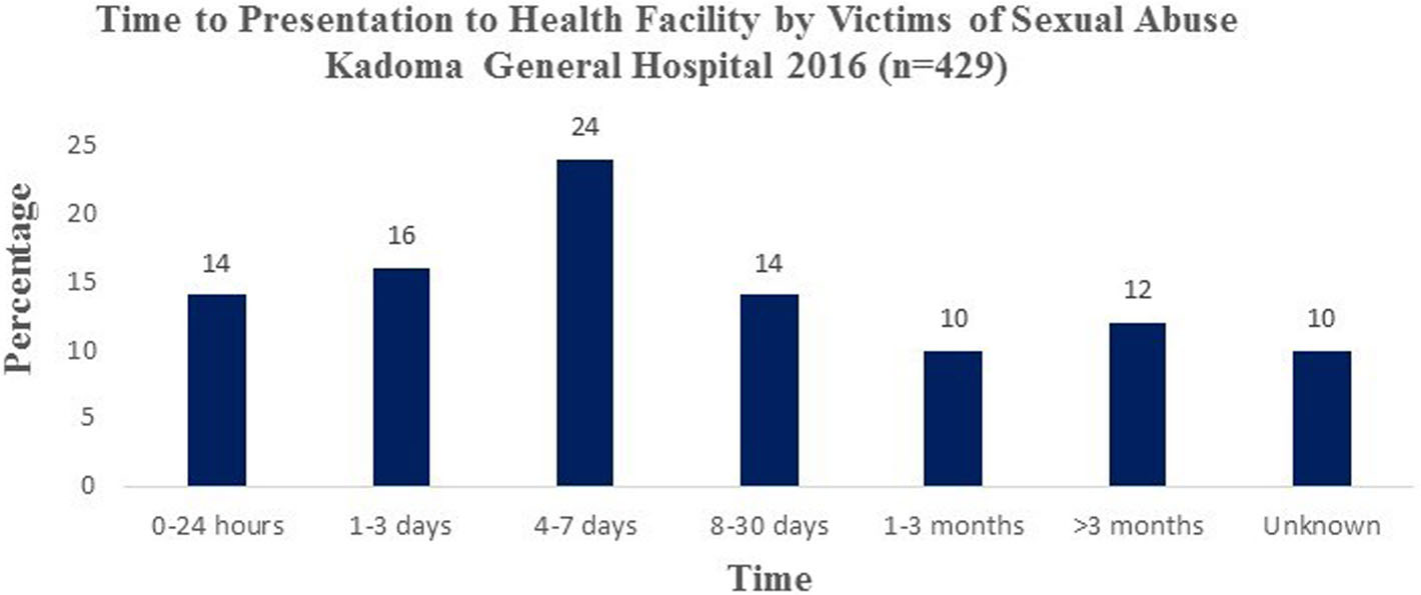
Time to presentation to the health facility by victims post sexual assault Kadoma General Hospital 2016

**Fig. 3:**
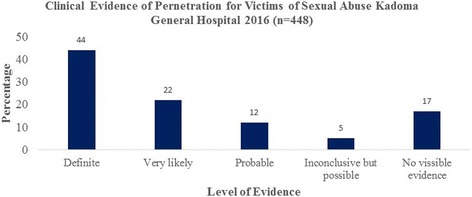
Clinical evidence of penetration for victims of sexual assault Kadoma General Hospital 2016

Time to presentation among victims of sexual assault is shown in Fig. 2. Thirty percent of the victims presented to the health facility within 72 h of being sexually assaulted. Twenty-four percent presented within four to seven days of being sexually assaulted whilst 154 (36%) presented after seven days and in 43 (10%) the time of the sexual assault was not known. Definite clinical evidence of penetration was found in 197 (44%), very likely 99 (22%), probable 54 (12%), inconclusive but possible in 22 (5%) and no visible evidence in 76 (17%) of victims as illustrated in Fig. 3. Condom use by the perpetrator of sexual violence was documented in 9 (2%) of sexual assaults.

### Clinical signs and symptoms in victims

**Fig. 4:**
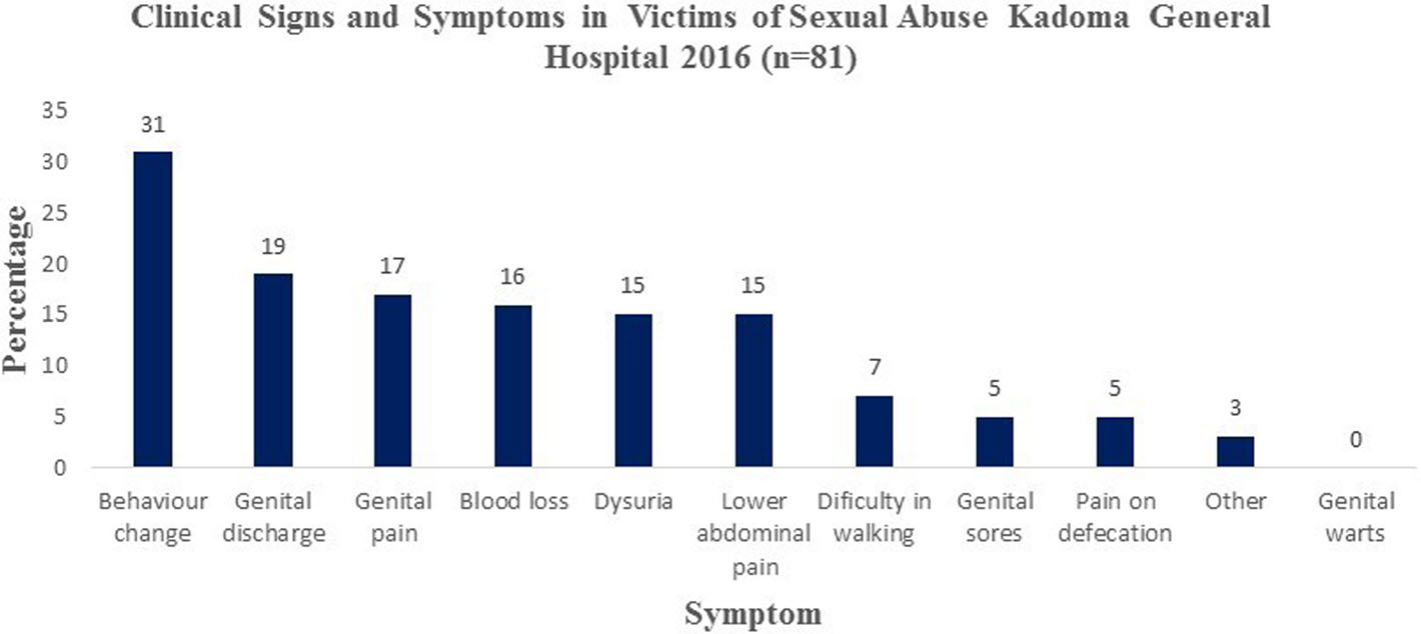
Signs and symptoms among victims of sexual assault Kadoma General Hospital 2016

External body injury was recorded in 185 (39%), anal injury 128 (27%), external female genitalia injury 182 (42%) and internal vaginal injury in 182 (42%). Among other clinical findings were behaviour change in 25 (31%), genital discharge 15 (19%), blood loss 13 (16%), dysuria 12 (15%) and lower abdominal pain 12 (15%). Genital sores and pain on defecation were documented in 4 (5%) victims as shown in Fig. 4.

### Clinical management in sexual abuse victims

Baseline HIV testing was done in 23 (22%) and second HIV test done in 2 (2%) of the victims. Ninety-eight (94%) victims were HIV negative on baseline HIV testing while six (6%) were HIV positive. Three victims out of eight who were initially HIV negative were found to be HIV positive on follow-up testing. Post-exposure prophylaxis for HIV was given to 18 (51%) and emergency contraception was given to 9 (69%) of the sexual assault victims presenting within 72 h of the sexual assault. Vaginal swab microscopy was done in one of the five victims who presented with a discharge. Prophylactic antibiotics were administered in 156 (33%) of the victims. Six out of 474 (1%) of the victims had forensic tests done. Of the 128 victims presenting within 72 h of the sexual assault, six (5%) had forensic tests done. Victims of sexual assault who had follow-up care were 47 (10%). Of all the victims attended at this institution there was no documentation of counselling sessions.

## Discussion

This was a cross-sectional study based on secondary data analysis. We sought to determine the clinical care given to victims of sexual abuse at Kadoma General Hospital, Zimbabwe between 2014 and 2016. The median age of victims in our study was 14 years. Sexual assault has been reported to be more common in this age group owing to the phase of sexual development that is adolescence [10]. This is also supported by the fact that the majority (40%) of the perpetrators in our study were boyfriends of the victims. However, our findings could be attributed to less reporting by older and married females owing to the fear of stigma and perceived social consequences associated with reporting sexual assault. In our study, females constituted 98.8% of the total victims of sexual assault. Our finding is consistent with that reported by Ige et al. [11] and Abdulkadir et al. [12] who reported a vulnerability of females to sexual assault owing to the patriarchal social norms that expose women to social injustice and exploitation. In our study, 74% of the perpetrators were known to their victims. We also found that the majority of perpetrators were boyfriends 81 (40%), relatives 46 (21%), stranger 51 (25%) and neighbour 15 (7%). Most of the victims fell prey to the people they knew unexpectedly, and this may be a result of the trust and relationship between the perpetrator that makes the victim assaulted unexpectedly. Girgira et al. [13] and Birdthistle et al. [14] reported similar findings in their studies in Ethiopia and Zimbabwe respectively.

In our study, the modal time to present to a health facility following sexual assault was between four and seven days. In Ethiopia, the median length of time taken to present to a health facility was found to be four days [13]. The delay in presentation to health facilities following sexual assault compromises medical interventions, as we expect victims to receive prophylactic therapy within 72 h of sexual assault to optimise the benefits. We found condom usage by perpetrators of sexual violence in 2% of the victims. The low condom usage results in a higher risk of contracting sexually transmitted infections and unwanted pregnancies among victims hence the need for optimum preventive interventions following sexual assault.

In a sub-analysis of 81 medical affidavits, genital discharge was recorded in 15 (19%) and bleeding in 13 (16%) of the victims. External body injury was reported in 185 (39%), anal injury 128 (27%), external female genital injury 182 (42%) and internal vaginal injury in 182 (42%). The presence of genital discharge and genital injury in these victims is substantial evidence of penetrative sexual assault. The presence of bleeding results in a breach in the mucosal surface of the genital tract hence predisposing the victims to acquire sexually transmitted infections. The importance of standard quality of care in victims of sexual assault cannot be underestimated. However, there could be more asymptomatic victims who later suffer sexual assault related complications such as subfertility and post-traumatic stress disorders. Vaginal lacerations with bleeding, hymeneal tear, and soft tissue injury have been reported in similar studies [11, 13].

In our study, prophylactic antibiotics were given in 36 (33%). However, one of the victims who had a vaginal discharge was not given antibiotics. Similar studies have also reported administration of post-exposure prophylaxis for sexually transmitted infections in 53.9% of the victims [13]. Ideally, prophylactic antibiotics should be given to all victims who present with or without symptoms of sexually transmitted infections as long as there is possible evidence of penetration. This prophylaxis is necessary to prevent the development of sexually transmitted infections and future sequelae associated with sexual assault [10].

The proportion of victims (30%) who were eligible to receive Post Exposure Prophylaxis (PEP) for HIV was low. This finding was due to delayed presentation for health care by victims following sexual assault. However, among those who presented on time, only 35 (51%) received PEP for HIV and 9 (69%) got emergency contraception. Owing to the services being provided in a busy outpatient department there is a possibility of missing some of the essential components of the clinical care delivered to victims of sexual assault. The victims also present to the hospital with a police officer whose priority is to have the medical affidavit signed for court proceedings and once the form is completed the victim is likely to leave the hospital without receiving the full package of care as required. There is need to prioritise the acute clinical care given to victims and this can be done by a multisector approach. Birdthistle et al. [14] in a study in Zimbabwe reported a high PEP coverage (92%) for victims presenting within 72 h [11]. The high PEP coverage found by Birdthistle owed to the model of service delivery at the health facility, in this case, it was a specialised clinic for sexual assault victims as compared to the outpatient model used at Kadoma General Hospital.

In our study, the proportion of victims who had baseline HIV testing was 22% which is low as we expect all victims to have a baseline HIV test. At baseline HIV testing, six (6%) of the victims were HIV-positive. Among these victims, three were adults who were sexually active but had never been screened for HIV infection before. Owing to the high HIV prevalence in Zimbabwe there is need to screen all victims for HIV as recommended by the guidelines. Of the eight victims tested for HIV at three months’ follow-ups, three became HIV-positive (38%). Missing PEP and appropriate care in the victims could have caused transmission of HIV in these three victims following the sexual assault. However, in settings with low HIV prevalence, the baseline and second HIV tests have been found to be negative in all victims [9, 13].

Our findings show that few victims were offered vaginal swab microscopy despite the overwhelming evidence of penetration and infection. Vaginal swab microscopy is essential for guiding antibiotic treatment in the victims with vaginal discharge. Despite 45 victims with definite evidence of penetration having presented within 72 h, forensic evidence was gathered in only six (13%) of the victims. Insufficiency of evidence during prosecution and lenient penalties for the perpetrators have resulted from the lack of adequate and convincing forensic evidence. Few of the victims (10%) in our study received follow-up care whilst there were no proper counselling sessions documented. This finding could be attributed to the non-existence of a proper follow-up mechanism for sexual assault clients. Counselling and follow up in sexual abuse victims has been shown to be suboptimal in several studies [11, 13]. Abdulkadir et al. [12] also reported non-documentation of the management of sexually transmitted infections, emergency contraception, HIV infection and scheduled follow up in a retrospective study of 81 sexual abuse victims in Niger State.

The major limitation encountered in our study was the incompleteness of variables relating to laboratory investigations and forensic tests and this could be attributed to the poor recording practices among clinicians. The insufficiency of the dataset could also affect generalisation of our study findings.

## Conclusions

We conclude that suboptimal clinical care was given to victims of sexual abuse during the period 2014-2016. These findings suggest possible delayed presentation by victims of sexual abuse as well as suboptimal administration of prophylaxis by health care workers. We recommend adherence to guidelines in managing sexual abuse victims. Further research to determine factors for delayed presentation for care among sexual abuse victims and quality of care provided to them is recommended.

## Abbreviations

GBV: Gender-Based Violence
HIV: Human Immunodeficiency Virus
PEP: Post Exposure Prophylaxis
STI: Sexually Transmitted Infections
ZDHS: Zimbabwe Demographic and Health Survey
